# Veterinary Forensic Pathology in the Investigation of Animal Cruelty: Post-Mortem Insights, Forensic Tools, Case Studies, and Legal Perspectives

**DOI:** 10.3390/ani16050785

**Published:** 2026-03-03

**Authors:** Julia Francesca Gilbert, Julia Eylül Aysu, István Tóth, Anna Szilasi, Míra Mándoki

**Affiliations:** 1 Department of Pathology, University of Veterinary Medicine Budapest, István utca 2, 1078 Budapest, Hungary; gilbert.julia@univet.hu (J.F.G.); eylulaysu@gmx.de (J.E.A.); szilasi.anna@univet.hu (A.S.); mandoki.mira@univet.hu (M.M.); 2 Department of Physiology and Biochemistry, University of Veterinary Medicine Budapest, István utca 2, 1078 Budapest, Hungary

**Keywords:** veterinary forensic pathology, animal cruelty investigation, forensic necropsy, post-mortem interval, forensic imaging, forensic entomology

## Abstract

Veterinary forensic pathology is an evolving discipline at the intersection of veterinary medicine, forensic science, and animal welfare law. This article underscores the growing importance of this field in advancing both animal welfare and justice. Drawing on post-mortem examinations, advanced forensic techniques, and real-world case studies, the article highlights how forensic veterinarians differentiate between ante- and post-mortem injuries, estimate time of death, and interpret complex trauma patterns. It also reviews the application of imaging technologies, bloodstain pattern analysis, forensic entomology, and histological tools in forensic investigations. Through illustrative case examples, including neglect, sexual abuse, thermal injuries, firearm wounds, and asphyxia, the article demonstrates the evidentiary value of veterinary pathology in legal contexts. Finally, the article outlines future directions for the field, emphasizing the need for standardized training, interdisciplinary collaboration, and greater legal recognition.

## Simple Summary

Animal cruelty is a widespread and often hidden problem that affects countless animals worldwide. This article explores how veterinary forensic pathology, a specialized field that applies medical science to legal investigations, can help uncover the truth behind suspicious injuries and an animal’s death. Through post-mortem examinations, veterinary experts can determine whether an animal suffered abuse, how and when it died, and whether the injuries were inflicted before or after death. The article also highlights the use of advanced tools such as imaging technologies, blood pattern analysis, and insect activity to support these investigations. Through real-life case examples, it demonstrates how forensic findings have been used to expose neglect, violence, and other forms of cruelty. In addition, the article discusses prospective advancements in the field, prioritizing the establishment of standardized training protocols, fostering interdisciplinary collaboration, and advocating for enhanced legal recognition. By combining scientific expertise with legal frameworks, veterinary forensic pathology plays a vital role in ensuring justice for animals and promoting a more humane society.

## 1. Introduction

Animal cruelty remains a pervasive global issue, affecting countless domestic and farm animals each year. Abuse, ranging from neglect and violence to organized fighting and sexual exploitation, often goes undetected due to the victims’ inability to communicate their suffering. In this context, veterinary professionals play a vital role in identifying signs of cruelty and advocating for animal welfare. However, the complexity of abuse cases demands more than clinical observation; it requires forensic science application to uncover the truth behind injuries and deaths.

In many jurisdictions, animal welfare organizations and law enforcement agencies report a growing reliance on veterinary post-mortem examinations in cruelty investigations. These cases frequently present practical challenges, including delayed case referral, incomplete crime scene documentation, and limited access to trained veterinary forensic specialists. As a result, necropsy findings often represent the primary source of objective evidence available to support investigative and legal decision-making.

Recent years have also seen rapid expansion of veterinary forensic medicine outside traditionally established centers in the United States and the United Kingdom, with growing contributions from South America, Europe, Asia, and the Caribbean, reflecting increasing international recognition of the field’s legal and societal importance [1].

Veterinary forensic pathology is situated at the intersection of medicine, law, and applied science, enabling systematic post-mortem examinations to distinguish injury timing, estimate time of death, and interpret trauma patterns with legal relevance. These insights are essential for understanding the circumstances of death and supporting legal accountability.

Despite its growing relevance, veterinary forensic pathology remains underutilized due to limited training, accreditation, and legal integration. Accurate post-mortem interpretation, supported by advanced forensic tools and interdisciplinary collaboration, is therefore essential to strengthen its role.

This article synthesizes current practices in veterinary forensic pathology, focusing on post-mortem techniques, forensic tools, and case applications, while outlining future directions for improving standards and legal recognition. By integrating scientific rigor with legal relevance, veterinary forensic pathology holds transformative potential in the fight against animal cruelty.

## 2. Post-Mortem Pathology in Veterinarian Forensics

### 2.1. Post-Mortem Changes and Interval Estimation

In post-mortem examinations, it is essential to understand the natural changes that occur after death. These physicochemical processes begin immediately or shortly after death, regardless of the cause, and follow a predictable sequence. However, their progression is heavily influenced by both internal and environmental factors, such as high temperatures, humidity, thick fur or fat layers, fever, or ante-mortem bacteremia [2]. Therefore, veterinarians and pathologists must be well-versed in these natural post-mortem changes to accurately distinguish them from potential indicators of animal cruelty or abuse, thereby avoiding misinterpretation. Moreover, recognizing and understanding these changes is critical for estimating the post-mortem interval (PMI), a key element in many forensic investigations. Accurate PMI estimation can help support or eliminate suspects and establish a timeline in suspected cases of animal abuse. Nonetheless, species-specific differences and methodological variability present challenges, making PMI estimation in animals more complex and less frequent than in humans [3].

Cadaveric decomposition is commonly divided into four main stages: fresh, bloat, active decay, and dry (or skeletal) remains. The fresh stage is characterized by autolysis and minimal external change. The bloat stage results from gas accumulation due to putrefaction, causing distension of the body. Active decay is marked by rupture of the body wall, release of gases, and extensive tissue breakdown with strong insect activity. The dry stage occurs after most soft tissues have decomposed, leaving desiccated tissue, cartilage, and bone. Recognition of these stages assists in contextualizing post-mortem changes and supports estimation of the post-mortem interval [4].

Typical post-mortem changes include algor mortis, rigor mortis, livor mortis (pallor and cruor mortis), desiccation, autolysis, putrefaction, and, under specific environmental conditions, mummification or adipocere formation. The onset and progression of these processes are influenced by environmental conditions, body condition, and pre-mortem factors, and must be interpreted in context during forensic evaluation [2]. When accurately interpreted, post-mortem changes can help veterinarians and forensic experts determine the possible cause of death.

#### 2.1.1. Thermal and Heat-Related Injuries

Thermal and heat-related injuries in animals may result from direct exposure to flames, hot surfaces, or liquids, as well as from radiant heat, electricity, microwaves, hyperthermia, or hypothermia. Localized burns are typically caused by direct or radiant heat, whereas systemic effects such as hyperthermia (heatstroke) and hypothermia produce more generalized tissue damage. The forensic evaluation of such injuries requires consideration of the cause, timing, and context, along with appropriate differential diagnoses.

In cases of burns, the shape and pattern of lesions can help identify intentional harm. As noted by Munro and Munro, “biologically abnormal patterns such as straight or angular borders, drip configurations, or unusual symmetry” may suggest deliberate burns [5]. For example, cigarette burns on companion animals often appear as circular, non-progressive lesions on the forehead, forelegs, or paws. Burns are classified by depth and severity [6]:

First-degree: superficial, affecting only the epidermis.
Second-degree: involving the epidermis and part of the dermis.
Third-degree: full thickness, affecting all skin layers, muscles, and tendons.
Fourth-degree: carbonization extending to underlying tissues.

Thermal damage may also extend to deeper tissues, including bone. Radiographic imaging is vital for distinguishing fractures caused by trauma from those due to heat exposure. Bones subjected to high temperatures often appear grayish-white and brittle, with a fine, network-like pattern of fractures visible on radiographs [7]. Determining whether an animal was alive at the onset of a fire is critical: minimal external injuries accompanied by smoke inhalation often indicate death from suffocation rather than burning. Microscopic signs of inflammation can support, but not definitively confirm, ante-mortem burns, as post-mortem heat exposure may produce similar lesions. (As discussed in Section 2.2, distinguishing ante- from post-mortem changes remains a central consideration in forensic pathology.) During handling and transport, burned cadavers must be protected from contamination, particularly the introduction of debris into airways [5].

Heatstroke, a systemic thermal injury, results from an imbalance between heat production and dissipation, leading to severe hyperthermia, inflammation, and multi-organ failure. It may be classic (environmental exposure) or exertional (intense activity in heat). In dogs, this condition most often occurs when confined in overheated vehicles or exercised in hot, humid conditions [8]. Temperatures exceeding 41 °C can cause neurological damage, above 41.5 °C induce apoptosis, and beyond 42.8 °C disrupt enzyme activity and membrane stability [9].

As ambient temperature and humidity rise, panting becomes less effective, trapping heat and increasing core body temperature. This positive feedback loop rapidly progresses to multi-organ dysfunction. Heatstroke shares several features with sepsis, including a cytokine-mediated inflammatory response that triggers endothelial activation, coagulation abnormalities, and tissue hypoxia. Common complications include renal failure, encephalopathy, and disseminated intravascular coagulation. Protective heat-shock proteins (HSPs) stabilize cellular structures and limit oxidative stress, but inadequate acclimatization or genetic factors can reduce their production, increasing susceptibility [10].

#### 2.1.2. Firearm Injuries

Firearm injuries in animals are commonly associated with three main types of weapons: air-powered guns, shotguns, and rifles. Each produces distinct injury patterns. Air-powered guns typically discharge single pellets with limited energy, resulting in minor damage. Shotguns release multiple pellets simultaneously, creating a scatter pattern of small wounds with relatively limited tissue damage. Rifles fire high-velocity bullets that cause deep and severe tissue trauma [5]. Gunshot wounds may be misidentified as puncture, bite, or laceration injuries [5], making it essential to accurately distinguish entry from exit wounds. Entry wounds usually have clean edges and may contain gunshot residue, while exit wounds tend to be irregular and jagged [11].

**Figure 1:**
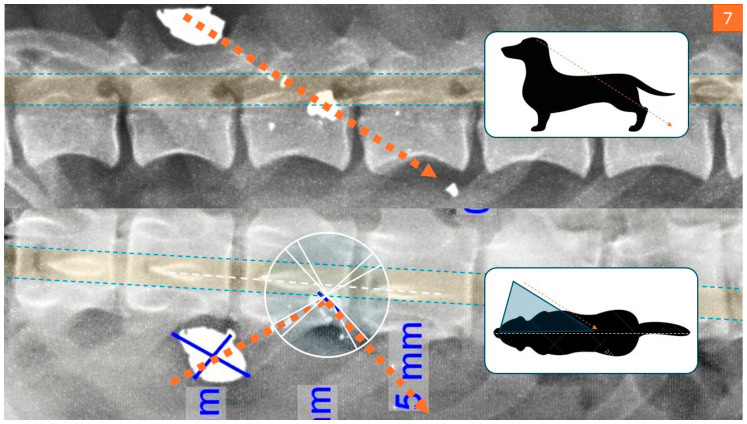
Bullet trajectory reconstruction derived from spinal X-ray imaging and final impact point analysis to determine the sequence of events. (Courtesy of Lorászkó Gábor, DVM).

Radiographic imaging is crucial when evaluating animals with unexplained injuries. It can reveal signs of gunshot trauma (Figure 1), such as fractures, pneumothorax, hemothorax, peritonitis, or retained projectiles, especially in joints, which may cause lead poisoning [7]. Imaging also helps locate pellets or bullet fragments and trace the projectile’s trajectory. High-velocity bullets often fragment on impact, producing a “lead snowstorm” pattern visible on radiographs [5], whereas low-velocity bullets break into larger, more identifiable pieces [5].

Kinetic energy is a key factor in assessing the extent of tissue damage. Radiographs also help differentiate exit wounds caused by fractures from those created by bullets. Familiarity with various weapons and ammunition types is therefore essential for accurate forensic evaluation [11].

#### 2.1.3. Asphyxia and Drowning

Asphyxia refers to impaired oxygen intake or carbon dioxide elimination, ultimately leading to cerebral hypoxia and cardiac arrest [5]. Common forms include suffocation, strangulation, traumatic asphyxia, and drowning. In forensic investigations, understanding the mechanism of oxygen deprivation is essential, as asphyxia often leaves no specific lesions [12].

Asphyxiation can occur through several means. Suffocation involves smothering or choking, while strangulation includes hanging, ligature strangulation, or manual compression of the neck. Mechanical asphyxia refers to restricted respiratory movements due to body positioning or external chest compression [7]. Although lesions such as petechiae, pulmonary edema, and congestion are frequently observed, they are nonspecific. Therefore, contextual evidence, such as ligature marks, foreign objects, and environmental clues, is critical for accurate diagnosis.

Drowning is defined as respiratory impairment resulting from submersion or immersion in liquid, resulting in death [13]. Forensic evaluation focuses on determining whether submersion occurred before or after death. Key findings may include distended, non-collapsing lungs; pulmonary edema and congestion; and frothy fluid in the airways, trachea, or nasal passages, suggesting ante-mortem respiratory effort. Water or foreign material in the stomach and respiratory tract further supports this interpretation. Microscopically, fluid-filled alveoli, intra-alveolar hemorrhages, and aspirated debris such as plant matter can corroborate drowning [14].

The diatom test is a valuable tool for confirming drowning by detecting diatoms, microscopic algae present in water, within the tissues of a deceased animal. Their presence in organs such as the liver or kidneys indicates ante-mortem aspiration of water [15]. Environmental conditions can also modify post-mortem changes: cold water may delay decomposition, while aquatic scavengers or environmental trauma can obscure lesions [14]. In drowning cases, the animal’s coat is typically wet, although perpetrators may attempt to dry the body to conceal evidence. Bruising from restraint or struggling may also be present [2].

In cases of live burial, multiple forms of asphyxia may contribute to the animal’s death. The animal may inhale or ingest soil, which can be found in the upper and/or lower airways, indicating active respiratory effort or ingestion prior to death. Additional signs such as broken claws with embedded soil and other evidence of struggle may be present, both on the body and within the burial site [7].

#### 2.1.4. Trauma Pattern Interpretation

**Figure 2:**
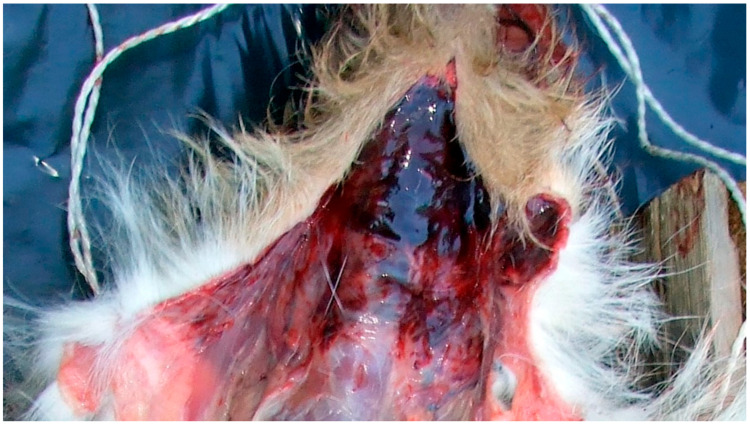
Hemorrhage and edema on the ventral aspect of the neck resulting from ligature strangulation with a white cord (Courtesy of Lorászkó Gábor, DVM.).

In cases involving trauma, differentiation between accidental and intentional injury is a central forensic challenge. Blunt force trauma (BFT), such as that resulting from vehicular impact or falls, may produce characteristic fracture patterns and soft tissue injuries that can overlap with those seen in intentional assault. Careful evaluation of injury distribution, multiplicity, symmetry, and associated environmental findings (Figure 2) is therefore essential to distinguish accidental from inflicted trauma.

Similarly, sharp force trauma requires assessment of wound morphology, margins, depth, and associated tissue bridging to determine whether injuries are consistent with animal activity, environmental hazards, or intentional use of implements. Integration of scene findings, necropsy results, and tool mark characteristics strengthens interpretation and reduces the risk of misclassification [7].

#### 2.1.5. Neglect

**Figure 3:**
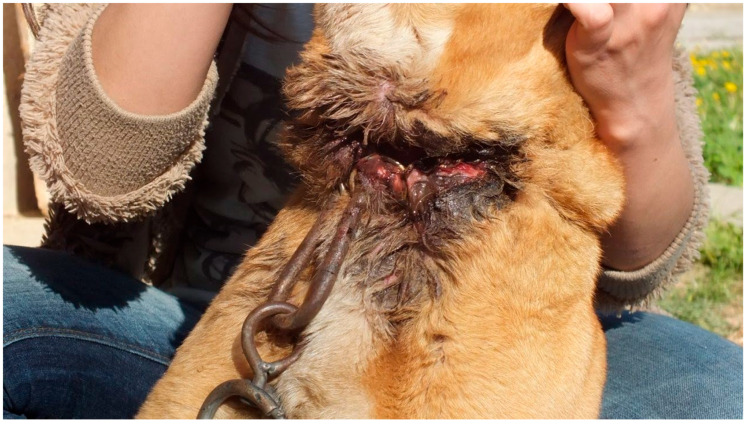
Severe circumferential cervical soft-tissue trauma consistent with chain-induced injury (Courtesy of Lorászkó Gábor, DVM.).

Neglect is often characterized by systemic deterioration and environmental indicators that reveal prolonged deprivation or inadequate care (Figure 3). Post-mortem examinations typically show changes consistent with starvation, dehydration, and poor hygiene, frequently accompanied by entomological evidence (larvae and adults). A study by Lutz et al. [15] identified key forensic markers such as pressure sores, malnutrition, dehydration, fecal contamination, and insect activity, particularly infestation by the house fly (*Musca domestica*). The development stage of larvae (myiasis) can help estimate the duration and severity of neglect.

Physical signs include decubitus ulcers, inflammatory skin lesions, and a noticeable loss of subcutaneous fat causing dehydration and skin wrinkling. A dull or brittle coat may indicate protein deficiency, while vitamin or mineral deficiencies, for example, zinc deficiency causing parakeratosis in pigs, provide additional diagnostic clues [5]. These findings are often accompanied by evidence of unsanitary living conditions. Forensic evaluations must distinguish neglect-related lesions from those resulting from natural post-mortem changes and consider any underlying medical or environmental factors [15].

Muscle and organ changes develop rapidly in neglected animals. Muscle atrophy may appear within 24 h of starvation, beginning in the back and thighs and later affecting all muscle groups. In severe malnutrition or prolonged suffering, rigor mortis may be delayed or incomplete due to depleted glycogen reserves [5]. When trauma or other stressors are also present, the pattern of rigor mortis can vary, underscoring the need to interpret such findings alongside case context rather than in isolation.

In emaciated animals, the stomach may contain fibrous or indigestible material, which can falsely suggest adequate feeding. Dogs and cats sometimes ingest non-nutritive items such as plastic, a behavior known as pica. These carcasses also tend to emit less odor than well-nourished individuals [5]. During prolonged emaciation, major organs shrink, especially the liver, spleen, pancreas, thymus, and salivary glands. In young animals, lymph nodes may appear enlarged due to reactive changes, and radiographs may show growth-arrest lines in long bones, indicating chronic malnutrition [5].

Underlying medical conditions, such as neoplasia, parasitic infestations, or chronic infections like Johne’s disease, must also be assessed to determine whether poor body condition results from illness rather than neglect [5].

#### 2.1.6. Toxicology

Forensic toxicology is a critical component of many animal cruelty investigations, particularly in suspected cases of intentional poisoning. Collection of appropriate samples, including gastric contents, liver, kidney, urine, blood, and bait materials available, is essential to distinguish intentional intoxication from accidental exposure. Interpretation must consider species-specific metabolism, environmental sources, and access to toxic agents. Proper chain of custody and storage conditions are necessary to preserve analytical integrity and legal admissibility [1].

### 2.2. Misleading Interpretations

In forensic investigations of animal cruelty, accurate interpretation of post-mortem changes is essential. These findings help determine the cause and manner of death, identify signs of abuse, and assess whether the animal experienced suffering prior to death. Misinterpretation of post-mortem changes can result in critical evidence of cruelty being overlooked or misclassified, potentially leading to incorrect conclusions and misleading the court and other stakeholders [5]. Such errors often arise from environmental or pathological factors that mimic ante-mortem injuries [16].

A well-structured post-mortem report is vital for distinguishing between injuries sustained before death and changes that occurred afterward. External signs such as bruising or fractures may be visible, but internal examinations can reveal hidden trauma, chronic malnutrition, or other indicators of prolonged abuse. Thorough documentation and clear categorization of findings are crucial for accurately reconstructing the circumstances surrounding the death [5]. Moreover, forensic necropsy reports serve as foundational legal evidence in cases of animal cruelty. These reports must be objective, detailed, and scientifically sound, as courts rely on them to evaluate the likelihood and extent of abuse [6]. Mistakes in determining wound age, bruise development, drowning status, or time since death can significantly distort the interpretation of events, potentially undermining justice [17].

Distinguishing between ante-mortem and post-mortem wounds can be challenging. Certain cellular responses, such as minor infiltration by inflammatory cells, may still occur in post-mortem injuries, potentially leading to misinterpretation. Additionally, wound healing timelines vary significantly between animal species, making it unreliable to extrapolate data from human studies. Even closely related species, such as dogs and cats, exhibit different healing rates [17]. Autolysis in deceased animals can also cause skin and hair to detach during handling, which must not be mistaken for ante-mortem trauma. Furthermore, bodies buried in shallow graves may be inadvertently punctured by search tools during recovery, creating post-mortem artifacts [18].

A study conducted at the University Hospital of Schleswig-Holstein in Kiel, Germany, demonstrated that mast cell analysis at wound margins can aid in differentiating ante-mortem from post-mortem injuries [18]. In living tissue, mast cells—along with other white blood cells—accumulate at injury sites to initiate healing. These cells release enzymes that support tissue repair, but once degranulated, they lose the ability to produce chloroacetate esterase, rendering them undetectable with specific stains. As a result, ante-mortem wounds often show fewer visible mast cells at the wound edge. In contrast, post-mortem wounds, which lack an inflammatory response, exhibit a uniform distribution of intact mast cells, indicating the injury occurred after death.

Estimating the age of bruises in animals is inherently complex due to species-specific differences in bruise development and healing. While bruise color and consistency change over time, these changes are highly variable and influenced by environmental conditions and the animal’s physiological state. Misinterpretation of bruise age can lead to significant errors in determining when an injury occurred, an essential factor in cases of suspected abuse [17].

A case study involving a dog euthanized after sustaining a bite injury illustrates this challenge. Photographs taken immediately after euthanasia documented the bruises, but during the subsequent autopsy, changes in skin coloration and the appearance of bruising became more pronounced. These post-mortem developments could easily be mistaken for ante-mortem injuries. This case highlights the unreliability of using bruise appearance alone to determine wound age after death [18].

Determining whether an animal was alive at the time of submersion in drowning cases is particularly challenging due to the lack of definitive forensic markers. Most signs of drowning are non-specific, and histopathological changes in the lungs do not offer the diagnostic certainty required for legal proceedings. Diatom testing, while commonly used, can produce misleading results due to contamination or false positives from post-mortem submersion, making it unreliable without corroborating evidence [17].

In neglect-related cases, post-mortem findings can be deceptive. Skin lesions such as pressure sores and tissue breakdown from prolonged immobility may resemble traumatic injuries. Parasitic activity, occurring both ante- and post-mortem, can mimic advanced decomposition or unrelated tissue damage. For example, maggot activity can alter wound margins or create openings in the skin, complicating the differentiation between neglect-induced injuries and external trauma. To address these challenges, forensic experts must integrate environmental, pathological, and entomological evidence to accurately reconstruct the circumstances of death [16].

Estimating the post-mortem interval (PMI) is further complicated by species-specific variability in decay processes. Methods based on body temperature or rigor mortis can yield inconsistent results across different species. Environmental factors such as temperature and humidity also influence decomposition rates, adding unpredictability. Entomological evidence can be valuable but requires species-specific expertise to interpret accurately. Without cross-verification, reliance on any single method may mislead forensic investigations [17].

Given these complexities, caution and species-specific knowledge are essential to avoid misinterpretation. Forensic pathologists must recognize the limitations of available techniques and adopt a multidisciplinary approach to improve diagnostic accuracy. This is especially critical in suspected abuse cases, where precise timing and injury interpretation can significantly influence legal outcomes [17].

## 3. Forensic Tools and Techniques

### 3.1. Crime Scene Assessment and Evidence Management

A forensic necropsy often begins at the crime scene rather than on the examination table. However, veterinarians responsible for necropsies are rarely present during the initial investigation [3]. A crime scene is not necessarily the location where the offense occurred, but any site where relevant evidence may be found and that serves as a reference point for legal proceedings [18]. Initial scene assessment provides essential context for interpreting necropsy findings, including the animal’s condition, environmental context, and situational indicators such as ambient temperature or the presence of bodily fluids [19].

Proper identification, collection, and preservation of evidence are critical to maintaining evidentiary integrity and preventing misinterpretation. Although forensic veterinarians are not expected to be experts in all forensic disciplines, most cases benefit from a multidisciplinary approach involving consultation with specialists such as toxicologists, entomologists, and osteologists. Recognizing professional limitations and seeking appropriate expertise is a core principle of forensic practice [3].

A structured approach to evidence management is required to ensure admissibility in court. This includes standardized photography, documentation of the animal and surroundings, minimal handling of the body, and appropriate packaging to prevent contamination. The use of forensic rulers and standardized imaging techniques supports accurate documentation of injuries and lesions [19]. In cases involving euthanasia, the method used must be recorded as post-euthanasia changes may mimic abuse-related injuries. Medical devices and prior wound evidence should remain in place to avoid misinterpretation [7].

Appropriate handling, including correct freezing and thawing procedures, is essential to preserve forensic evidence. Improper storage can obscure critical indicators such as hemorrhage, reduced bone marrow fat, or toxicological markers, thereby compromising interpretation and legal reliability [7].

When feasible, thorough scene examination may reveal indicators of restraint, struggle, or attempted escape. In cases involving multiple deceased animals, body positioning and condition may assist in determining whether deaths occurred simultaneously or over time, which is particularly relevant in suspected neglect cases [5]. Crime scenes may be classified as: (1) incident, death, and discovery at a single location; (2) incident at one location with death occurring elsewhere; or (3) incident and death at one location with subsequent body transport [5].

In neglect and starvation investigations, the primary objective is to document physical condition and assess whether concurrent disease contributed to the findings. Common indicators include poor hygiene, overgrown nails or hooves, dental abnormalities, poor body condition, and evidence of parasitism [20]. In cases of severe matting or parasitism, hair coat and parasites should be preserved as evidence, and targeted examination of skin, hooves, paw pads, and dentition should be performed and documented [20].

Body condition provides a key forensic indicator of nutritional status. Severe neglect results in emaciation characterized by depletion of fat reserves and muscle wasting. Internal examination should assess fat stores in the omentum, kidneys, pericardium, and bone marrow, and the gastrointestinal tract should be examined to document ingested material [20]. Standardized body condition scoring and detailed photographic documentation are essential. The estimated ideal body weight should be compared with actual weight, and conclusions should address whether caloric intake was sufficient to maintain health and whether underlying disease could account for the observed condition [20].

### 3.2. Imaging and Virtopsy

Veterinary forensic pathology employs a range of advanced techniques to accurately assess cases of animal cruelty. Necropsy remains the cornerstone of this process and must be performed with the utmost precision, as it is a one-time opportunity to gather critical evidence. A comprehensive necropsy includes both external and internal examinations—documenting species, physical characteristics, and visible injuries, followed by a detailed assessment of internal organs to determine the cause of death [21].

**Figure 4:**
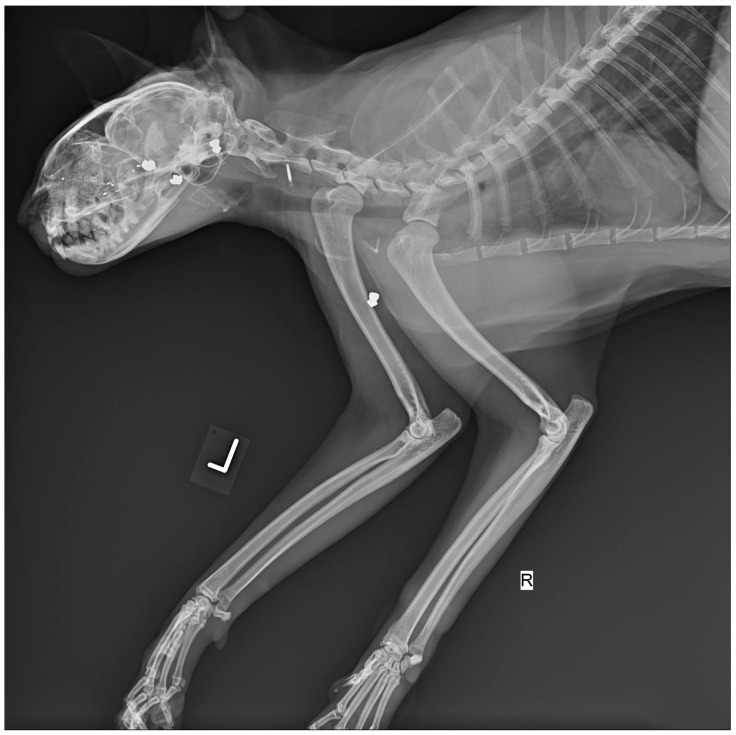
X-ray image of cat with multiple pellets lodged in the sinus cavity, chest and forelimb (Courtesy of Lorászkó Gábor, DVM.).

Modern imaging technologies, such as computed tomography (CT) and magnetic resonance imaging (MRI), significantly enhance forensic evaluations. While they cannot replace traditional necropsy, they offer valuable non-invasive insights. Traditional radiography (X-ray) has long been used to detect internal injuries, including fractures, embedded objects, and trauma. In shooting cases, for example, X-rays are a basic yet decisive tool (Figure 4). CT scans provide more advanced imaging, offering both 2D and 3D reconstructions that allow for precise identification of subtle injuries and foreign objects. When combined with angiography, CT imaging can also visualize the vascular system, which is particularly useful in cases involving circulatory trauma [21].

Forensic pathologists play a crucial role in interpreting these findings. Their expertise allows them to determine the cause and mechanism of injuries, and to distinguish between ante-mortem and post-mortem trauma—an essential distinction in suspected abuse cases. They also apply techniques from human forensic pathology, such as microbiological and toxicological analyses, to confirm causes of death [22].

An emerging tool in veterinary forensics is the virtual autopsy or virtopsy, which combines CT, MRI, photogrammetry, and laser scanning. Originally developed for human forensics, virtopsy is increasingly used in animals to capture detailed external and internal data without disturbing the body. Photogrammetry paired with laser scanning can produce high-resolution 3D color models of internal injuries, aiding crime scene reconstruction and linking injuries to specific objects or trauma types [21].

Despite its advantages, post-mortem imaging has limitations. It may not reveal critical diagnostic details such as tissue color or consistency, and certain soft tissue injuries—like cardiac or ischemic lesions—can be difficult to detect. Nonetheless, imaging offers significant benefits, including non-invasive sample collection for histological or toxicological analysis, reduced infection risk, and the ability to digitally preserve evidence for repeated review and expert consultation [21].

### 3.3. Bloodstain Pattern Analysis and Entomology

#### 3.3.1. Bloodstain Pattern Analysis in Forensic Contexts

Bloodstain Pattern Analysis (BSPA) is a forensic technique that applies principles from physics, biology, and chemistry to interpret bloodstains found at a crime scene. While grounded in science, BSPA also involves a degree in interpretation, making it both a scientific and evaluative discipline. It aids investigators in reconstructing events by analyzing the size, shape, distribution, and location of bloodstains. From these patterns, forensic experts can infer critical details such as the type of assault, the number of blows, the movement of the victim, and the positions of individuals involved [23].

**Figure 5:**
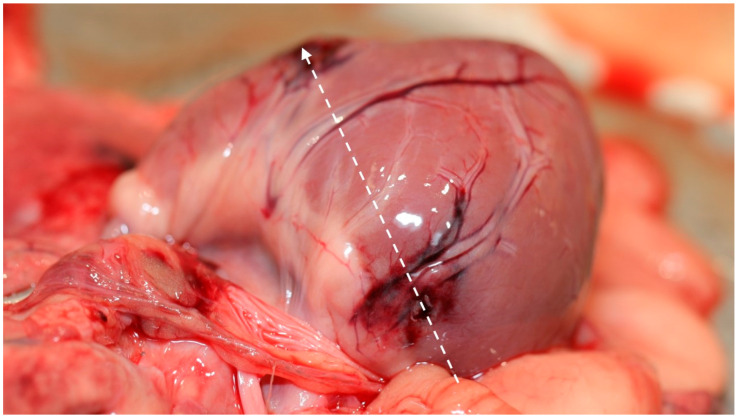
Gross image of the heart demonstrating the reconstructed cardiac bullet trajectory (dashed line), correlating the wound path with the source of arterial spray formation. (Courtesy of Lorászkó Gábor, DVM).

BSPA categorizes bloodstains into several types:

- Passive stains (e.g., drips and pools) result solely from gravity.
- Spatter patterns (e.g., from gunshots or blunt force trauma) are caused by forceful impacts (Figure 5).
- Altered stains are modified by external factors such as drying, smearing, or contact with surfaces.

Each category provides unique insights into the events that occurred [23].

A key component of BSPA is determining the area of convergence, which is the two-dimensional point where blood droplets originated. When extended into three dimensions, this becomes the area of origin, offering a clearer understanding of the height and position of the blood source. Although BSPA may not always yield definitive conclusions, it is a powerful tool for supporting or challenging witness statements by comparing physical evidence with reported accounts [23].

Given the complexity of Bloodstain Pattern Analysis (BSPA) and the risk of contamination at crime scenes, it is essential that trained experts handle the documentation and collection of bloodstain evidence with great care. When limitations arise—such as overlapping patterns or altered stains, the most responsible approach is to avoid drawing unsupported conclusions. In forensic investigations, BSPA should be viewed as a supplementary tool that helps guide investigators toward the most plausible sequence of events based on the available blood evidence [23].

#### 3.3.2. Insect Colonization for Timeline Estimation

Forensic entomology has a long history, with early documented applications dating back to 13th-century China and later systematic developments in Europe and North America during the late 19th and early 20th centuries. These early observations established insects and other arthropods as valuable indicators in medicolegal investigations and laid the foundation for modern forensic entomology [24].

In forensic investigations, the presence of insects, particularly flies and beetles, can provide valuable information for estimating the time of death or the duration of neglect in both humans and animals. When flies colonize wounds or natural body openings in living individuals, the condition is known as myiasis, which is often associated with neglect. The development of these insects is highly temperature-dependent: warmer conditions accelerate their growth, while cooler environments slow it down. Arthropod activity, especially from blowflies, often begins before death in neglected individuals, leaving distinct patterns that forensic entomologists can analyze. The developmental stages of maggots, their distribution, and associated necrotic tissue provide critical information about the duration and extent of suffering. These findings, combined with post-mortem changes such as decomposition and tissue breakdown, contribute to comprehensive forensic assessments in both animal and human neglect cases [16,25].

Forensic entomology can be used to estimate both the minimum and maximum post-mortem interval (PMI). The minimum PMI (minPMI) is typically derived from the age and developmental stage of the oldest insect specimens present, reflecting the earliest possible time of colonization. In contrast, the maximum PMI (maxPMI) may be estimated using patterns of insect succession, in which different arthropod species colonize remains in a predictable sequence over time. Analysis of the entomofaunal succession pattern, in conjunction with environmental data, can therefore provide broader temporal context for estimating the duration since death or neglect [16].

In cases of animal cruelty or neglect, forensic entomology can offer critical evidence. Adult flies are often attracted to wounds or areas contaminated by bodily fluids, where they deposit their eggs. These sites become colonization points for maggots, which develop over time. During forensic investigations, it is essential to collect and preserve maggots from different body regions, especially from wounds, separately. This allows for the observation of variations in developmental stages, which can help estimate the time and location of insect colonization, thereby contributing to the reconstruction of the timeline of neglect or death [25].

A practical example of this application involved a two-year-old French Bulldog, whose owner claimed the dog had been alive and well the day before its death. Upon examination, the veterinary pathologist observed numerous fly maggots, nits in the fur, and early skeletonization of the right upper jaw. Additional findings included the absence of subcutaneous and internal fat normally found in the mesentery and an empty stomach. Veterinary authorities collected maggots from the body and analyzed them to estimate the minimum colonization period. By examining the developmental stage of the maggots and referencing local temperature data, forensic experts were able to approximate the timeline of colonization, offering insight into the timing of death and potential neglect [25].

**Figure 6:**
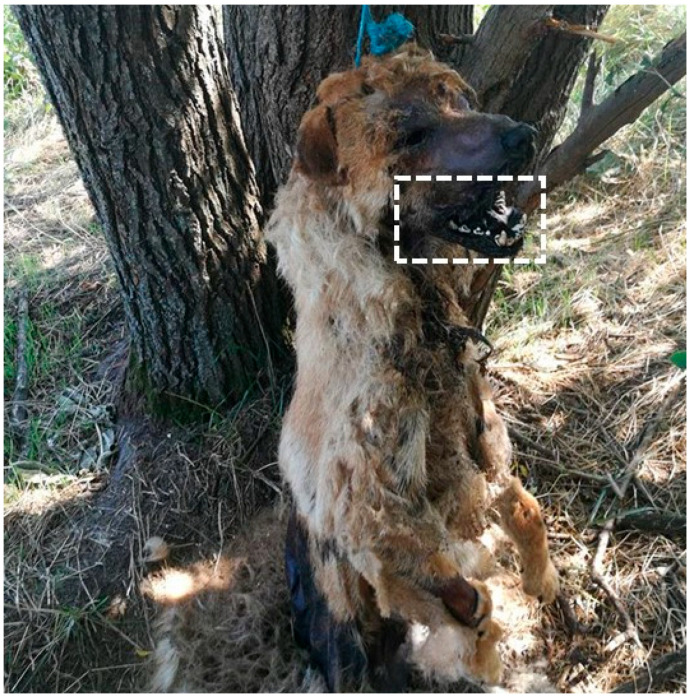
Post-mortem ocular loss resulting from dipteran larval feeding, with advanced decomposition affecting the oral cavity and fur (Courtesy of Lorászkó Gábor, DVM.).

The entomological analysis concluded that the dog could not have been alive and healthy the evening before, as claimed by the owner. The advanced decomposition, including severe brain degradation or absence, indicated that cadaveric fly colonization had begun at least two days prior (Figure 6). This estimate could be even earlier if the dog had been kept in cooler conditions than the recorded outdoor temperature of 30 °C [25].

This case underscores the importance of careful preservation and documentation of insect evidence by veterinarians and forensic pathologists. Proper handling of maggots and other entomological traces is essential, particularly in complex cases where precise timelines are critical for drawing reliable conclusions [25].

Standardized collection and documentation protocols are essential to ensure the forensic value of entomological evidence in animal cruelty investigations. Recommended procedures include systematic sampling of larvae and adult insects, collection from multiple body regions and surrounding substrates, preservation of specimens for both morphological and molecular analysis, and detailed recording of environmental conditions. Adherence to established protocols enhances reliability, reproducibility, and legal admissibility of entomological findings in cruelty cases [26].

### 3.4. DNA Evidence

DNA analysis is increasingly used in veterinary forensic investigations and represents one of the most powerful tools for associating animals, weapons, and potential suspects. DNA may be recovered from wounds, suspected implements, hair, saliva, or touch DNA transferred during handling or assault. These methods allow linkage of specific objects to injuries, identification of animals involved in bite or attack incidents, and differentiation between animal predation and human-inflicted trauma. Emerging research also demonstrates the utility of touch DNA in identifying human–animal contact, expanding the evidentiary potential in abuse investigations [7].

### 3.5. Neutrophil Migration After Injury

In forensic pathology, accurately determining the timing of an injury is vital for reconstructing events and understanding trauma. One effective method involves analyzing the behavior and spatial distribution of neutrophils—immune cells that respond rapidly to tissue damage. These cells exhibit distinct migration patterns within hours of an injury, making them reliable indicators for estimating the age of a contusion.

Advanced histological and immunohistochemical techniques are used to study neutrophil activity, which typically peaks within the first 24 h post-injury. Parameters such as their concentration, migration distance, and distribution patterns provide valuable clues about the timing and progression of trauma.

A controlled study [27] utilized digital pathology systems, including whole-slide imaging and computer-assisted image analysis, to quantify neutrophil migration near blood vessels in injured skeletal muscle. The results showed that neutrophils initially cluster near the injury site and gradually spread out over time. This precise tracking allows forensic pathologists to estimate injury timing with high accuracy, particularly during the critical early hours following trauma—applicable in both veterinary and human forensic cases [27].

### 3.6. Video Evidence

Video recordings offer critical, real-time insights into potential crimes involving animals and their behavior. These recordings may originate from various sources, including CCTV systems, law enforcement body cameras, ATM surveillance, dashboard cameras, or personal mobile devices. Once obtained, the footage undergoes detailed analysis to verify its authenticity, enhance visual clarity, and determine its relevance for expert testimony.

In suspected cases of animal cruelty, veterinarians may be asked to review such footage as part of a forensic investigation, though this is less common in animal-related cases. The value of video evidence lies in its ability to provide a unique perspective on animal behavior and treatment, potentially revealing signs of physical or emotional suffering. The ‘Five Freedoms’ framework, encompassing nutrition, comfort, health, normal behavior, and freedom from fear and distress, serves as a structured checklist for veterinarians to systematically assess animal welfare in video footage [23]. A structured approach is essential for a thorough and objective evaluation. This process typically involves five key steps:

Careful Review: Watch the video attentively to understand the context and events.

Assessment of Animal Characteristics: Evaluate physical and behavioral indicators such as body condition, posture, vocalizations, and mental state.

Identification of Abnormalities: Look for signs of poor health, fear, injury, inadequate shelter, or violations of the Five Freedoms.

Formulation of Expert Opinion: Summarize findings in a report or prepare for court testimony.

Presentation of Findings: Clearly communicate conclusions, supported by context from other investigative data.

In summary, video recordings are powerful tools in uncovering and documenting animal cruelty. Through systematic analysis, veterinarians can provide expert insights that are often pivotal in legal proceedings involving abuse or neglect [23].

## 4. Case Studies in Veterinary Forensics

Most published cases in veterinary forensic pathology originate from North America and Europe, particularly the United States, the United Kingdom, and Germany. Companion animals, especially dogs, are the most frequently studied species, followed by cats, livestock species, and wildlife. This reflects both reporting patterns and the close association of companion animals with human households, which increases detection and investigation of suspected cruelty [20].

### 4.1. Neglect: Chronic Illness Misinterpreted as Abuse

A 6.5-year-old cross-bred bull terrier, previously known to be subjected to physical abuse by its owner, was seized following renewed reports of mistreatment. Upon clinical examination, the dog presented with multiple deep, scabbed wounds on the head, bruising around the muzzle, and fractured canine teeth. Additional bruising was noted on the neck and left axillary region, accompanied by subcutaneous emphysema extending from the neck and thorax across the rib cage.

Radiographic imaging revealed multiple rib fractures and signs of bone degeneration in the femur and spine. Despite medical intervention, the dog’s condition deteriorated, ultimately leading to euthanasia on humane grounds.

Post-mortem examination identified multiple myeloma as the underlying condition. This malignancy had caused 14 rib fractures of varying ages, spinal collapse with associated spinal cord compression, and tumor infiltration in the femur and small intestine. While the rib fractures could not be definitively attributed to physical abuse, the findings highlighted a failure to diagnose and treat a progressive, painful disease. The court found the owner guilty of animal cruelty for neglecting to provide essential veterinary care.

Furthermore, the healing wounds on the dog’s head were consistent with burn injuries, potentially inflicted by a cigarette. Although histopathological analysis ruled out differential diagnoses such as autoimmune, parasitic, and fungal conditions, the available evidence was insufficient to support an additional charge of intentional cruelty [5].

### 4.2. Sexual Abuse: Forensic Confirmation Through Trauma and Digital Evidence

Law enforcement became involved when disturbing images were discovered during routine screening of digital image files submitted for photo processing. The images showed a person inserting a mallet handle into the vagina of a dog, along with photos showing human male genitalia in contact with a dog’s vulva.

Following the acquisition of a search warrant, authorities took two female Labrador Retrievers, aged approximately 3 to 5 years, from the suspect’s residence. Both dogs displayed significant evidence of vaginal trauma, with visible linear lesions extending over 10 cm into the vaginal area.

In court, law enforcement established the identity of the dog’s owner as the individual shown in the photographs. The prosecution’s approach focused primarily on demonstrating the extent of the torture, pain, and suffering of the animals rather than searching for the explicit details of the abuse itself. Eventually, a felony conviction was secured [4].

Investigations of suspected sexual assault require strict adherence to forensic protocols, including collection of swabs for semen, saliva, and touch DNA, documentation of genital and perianal trauma, and preservation of bedding, restraints, and clothing. Use of standardized sexual assault evidence kits and coordination with forensic laboratories enhances evidentiary value and supports legal proceedings, as detailed in established veterinary forensic guidelines [4].

### 4.3. Thermal Injuries: Ante-Mortem Burns Confirmed by Lung Analysis

In an act of intimidation by a criminal gang involved in serious crimes, a bull terrier belonging to a member of a rival was abducted. The dog was secured to a railing by its chain collar, cut and stabbed with a knife, and then set on fire.

The findings of the necropsy include the dog’s skin exhibiting a parchment-like texture over much of the body with the most severe charring affecting the head, shoulders, and the front of the chest. The subcutaneous muscles of the head and shoulders appeared tawny and partially cooked. The impression of the chain was deeply embedded in the burned skin of the neck. Furthermore, there were extensive lacerations and splits in the skin over the front of the chest, left shoulder, and the front surface of the left foreleg between the elbow and wrist. The frontal bone and the left zygomatic arch were fractured. A significant amount of blood had accumulated in the frontal sinuses, and there was fragmentation and loss of teeth in the left upper jaw. Lastly, in the respiratory tract, findings included blood pooling in the larynx and upper trachea, blood-stained froth in the upper trachea, moderate amounts of blood and mucus in the lower trachea and main bronchi, severe congestion and flooding of the lungs, and numerous inhaled carbon particles in the air sacs of the lungs.

The blood found in the frontal sinuses suggested that the skull fractures occurred before death. The presence of blood, froth, and carbon particles throughout the respiratory tract clearly indicated that the dog was alive while exposed to the fire.

The splitting of skin in burned bodies should be considered carefully, as heat can cause splits in the skin, particularly over extensor surfaces and joints, which may resemble ante-mortem injuries. This phenomenon, observed in human burn victims, could also apply to animals. Such splits must be distinguished from true wounds, which can be challenging when there is significant heat damage. Examining deeper tissues may provide definitive evidence of injuries inflicted before death [5].

### 4.4. Firearm Injuries: Entry/Exit Wound Analysis and Bullet Trajectory (Figure 7)

A pine marten (*Martes martes*) was discovered in a domestic freezer, exhibiting two primary areas of injury. Findings in the chest included significant blood staining on both sides of the chest. An eight-millimeter hole was found near the sternum on the lower right side, while a larger, irregular bigger hole was observed in the left chest wall between ribs four and five. Massive bleeding had filled both sides of the thorax, and extensive damage was noted in the middle sections of the lungs. Furthermore, head injuries were observed with an elliptical hole on the top of the head with hair pulled into it. The surrounding skin was bruised. A hole penetrated the skull, accompanied by an irregular fracture in the cranium. The left side of the skull showed multiple fractures, and a large hole was located at the angle of the mandible on the left side. Most of the brain was destroyed, and fragments of a bullet were found in the head, jaw, and skin near the jaw. There was no significant bleeding along the path of this bullet from the top of the head to the left side of the head. The investigation concluded that the pine marten had initially been killed by a rifle shot to the chest. A second shot was fired at the head shortly after death, likely to ensure that the animal was deceased [5].

**Figure 7:**
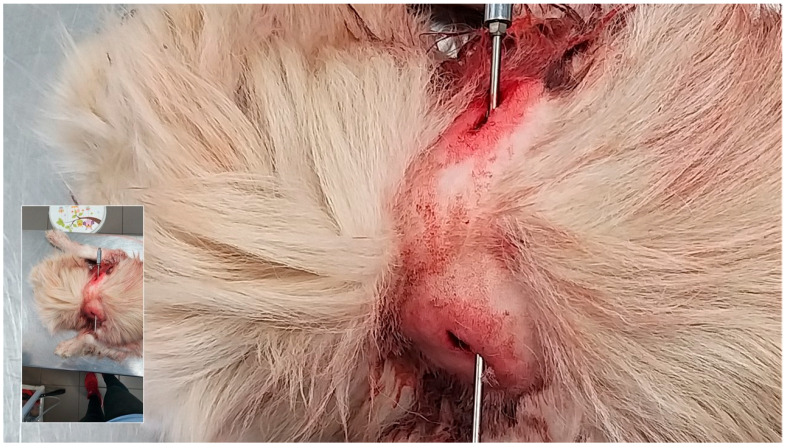
Gunshot wound to the cervical spine with probe-guided visualization of the projectile track (Courtesy of Lorászkó Gábor, DVM.).

### 4.5. Asphyxia: Ligature Marks and Absence of Smoke Inhalation

Firefighters responded to an apartment fire, primarily concentrated in the bedroom. After extinguishing the blaze, they searched for the remaining areas of the apartment. In the kitchen, they discovered “Taffy,” a middle-aged female dog, lying deceased in her bed.

Due to the suspicious circumstances surrounding the fire, which raised concerns of arson, the police requested a post-mortem examination of Taffy to determine if smoke inhalation was the cause of death.

The findings of this case revealed that Taffy was in good physical health, showing signs of proper care with no evidence of natural illness. However, a single subcutaneous bruise was observed on the back of her neck. Her lungs, trachea, larynx, eyes, and subcutaneous tissues were heavily congested, yet there was no evidence of smoke inhalation.

The findings suggested that Taffy had not died from smoke inhalation but from asphyxiation caused by a ligature around her neck.

Upon questioning, the owner’s boyfriend confessed to hanging Taffy using the cord from a dressing gown, suspending her from the loft hatch before setting the apartment on fire [5].

## 5. Future Challenges and Opportunities

Veterinary forensic pathology is progressing as a specialized field, reflecting its increasing importance in legal investigations involving animal cruelty. Unlike routine diagnostic necropsies, forensic examinations prioritize evidence preservation and adherence to legal standards. However, despite growing demand, many veterinary pathologists report limited formal training in forensic methodologies, creating a skills gap that can compromise the effectiveness of investigations and prosecutions [28].

Growth in veterinary forensic science has been driven in part by increased public and legal attention to animal cruelty, as well as recognition of the association between animal abuse and other forms of interpersonal violence. The development of professional networks and interdisciplinary infrastructure has expanded the field’s role in both animal welfare and broader public safety efforts [20,29].

In this evolving context, veterinary forensic pathology is incorporating advanced diagnostic approaches, including molecular and ancillary techniques, to enhance diagnostic accuracy and strengthen the scientific basis of forensic interpretation. These developments are most evident in regions with established forensic and veterinary infrastructure, highlighting ongoing international variability in access to training and resources [20].

Formal education and training programs in veterinary forensic science have begun to emerge, reflecting efforts to address existing capacity gaps. These initiatives aim to provide structured instruction in crime scene analysis, forensic necropsy, evidence documentation, and courtroom testimony. However, access to specialized training remains uneven, underscoring the need for broader international investment in education, accreditation, and capacity building [29].

Effective animal cruelty investigations increasingly require close collaboration between forensic veterinarians and veterinary pathologists. Integrated involvement, from scene investigation through necropsy, ancillary testing, and expert testimony, supports more accurate lesion interpretation and case reconstruction. Where responsibilities are divided, detailed case documentation, high-quality imaging, and interdisciplinary communication are essential to mitigate misinterpretation and loss of context [20].

Despite ongoing advances, veterinary forensic pathology continues to lack universally standardized protocols and consistent legal integration. Continued development of guidelines, training frameworks, and collaborative practice models is therefore essential to strengthen evidentiary reliability and promote consistent forensic standards across jurisdictions [20].

## 6. Conclusions

Veterinary forensic pathology is advancing rapidly [30], yet it continues to face structural limitations that hinder its full potential in addressing animal cruelty. While public awareness and demand for veterinary forensic services have grown, the field still lacks the standardized training, protocols, and medicolegal infrastructure that are well-established in human forensic sciences. This disparity often prevents veterinary pathologists from fully contributing to legal proceedings.

In contrast, human forensic pathology plays a central role in the justice system, offering scientifically robust evidence that frequently leads to accurate legal outcomes and accountability for perpetrators. However, such comprehensive forensic support is rarely available in animal cruelty cases. As a result, investigations often lack the evidentiary strength needed to determine what happened to an animal, allowing offenders to evade meaningful consequences. These limitations, as discussed above, highlight the ongoing need for stronger integration of veterinary forensic pathology into legal frameworks and investigative practices. The establishment and widespread adoption of standardized protocols for forensic necropsy, evidence collection, documentation, and reporting are essential to ensure consistency, reproducibility, and legal admissibility of findings. Harmonized procedures would strengthen the scientific credibility of veterinary forensic pathology and enhance its value within judicial systems.

## Data Availability

No new data were created or analyzed in this study. Data sharing is not applicable to this article.
